# “Do you see what I mean?” staff collaboration in eating disorder units during mealtimes

**DOI:** 10.1186/s12912-017-0233-3

**Published:** 2017-07-20

**Authors:** Trine Wiig Hage, Øyvind Rø, Anne Moen

**Affiliations:** 1 Institute for Health and Society, University of Oslo, Oslo, Norway; 2 Oslo University Hospital, Regional Department of Eating Disorders, Division of Mental Health and Addiction, Oslo, Norway; 3 Institute of Clinical Medicine, University of Oslo, Oslo, Norway

**Keywords:** Staff members, Eating disorders, Collaborative practice, Interviews, Cultural historical activity theory, Mealtimes

## Abstract

**Background:**

Eating disorders are psychiatric illnesses with potentially life-threatening consequences. Inpatient treatment is typically required for the most severely ill patients, who are often emaciated or significantly malnourished. A core therapeutic objective is to normalize eating patterns and facilitate weight gain. These goals guide the efforts of milieu therapeutic staff working with this patient group, who support renourishment through the positive manipulation of a structured environment, as well via relational aspects. However, there is a lack of empirical research exploring inpatient staff members’ perspectives concerning various aspects of this work. This article explore staff’s teamwork during mealtimes on inpatient eating disorder units. Specifically, we investigated the collaborative strategies employed to support core therapeutic goals of meal completion and normalized eating behavior, while concurrently maintaining a supportive, friendly atmosphere during mealtimes.

**Methods:**

This was a exploratory qualitative study. Data was collected through 20 semi-structured in-depth interviews with staff members working on a specialized eating disorder unit. The interviews were performed after the conduction of meal time support. Cultural historical activity theory was used as the key theoretical tool for analysis.

**Results:**

The analysis revealed three main themes: 1) strategic seating arrangements mediates division of labor, 2) the use of verbal and nonverbal communication as collaborative tools, and 3) the importance of experience as a collaborative resource.

**Conclusions:**

The present study found that mealtime collaborative strategies on inpatient EDUs were mainly of non-verbal nature, with level of experience as an important premise for staff collaboration. Greater awareness about how collegial collaboration is practiced may help staff members to learn routines and regulate scripts for mealtime practices.

## Background

Healthcare organizations are prime examples of collaborative, complex work settings. A key characteristic of these settings involves a strong reliance on each member’s understanding of and contribution to achieving specified goals, as well as effective teamwork and good communication [1]. This understanding often reflects operating standards, accumulated experiences and collective knowledge which, in turn, helps construct and consolidate in-house practices [2]. The reliance on staff’s understanding of shared knowledge and in-house procedures may vary across different clinical practice settings. For this study, the working context involves milieu therapy on a specialized eating disorders unit (EDU). Milieu therapeutic work on inpatient EDUs can be characterized as a highly specialized knowledge practice.

Eating disorders (ED) are classified as mental health disorders and comprise a group of severe illnesses that range from ‘moderate – severe’ through to ‘life threatening’. EDs are categorized in DSM 5 as Anorexia Nervosa (AN), Bulimia Nervosa (BN), Binge Eating Disorders and Otherwise Specified Feeding and Eating disorders [3]. Inpatient treatment is required for the most severely ill patients, typically those suffering from AN, who are often emaciated or significantly malnourished. A core therapeutic objective is to normalize eating patterns and facilitate weight gain [4]. These goals guide the efforts of milieu therapeutic staff, who support renourishment through the positive manipulation of a structured environment, as well via relational aspects [5, 6]. In ED treatment, the staff’s contribution to successfully attaining these treatment goals is of utmost importance, not least due to the considerable amount of time they spend with patients [7]. Daily care on EDUs is provided by nurses and other health care professionals, like social workers or child welfare officers, employed as members of the milieu therapeutic team (henceforth referred to as ‘staff’). High-quality relationships needed in an therapeutic environment requires knowledge about staff perspectives. However, there is a lack of empirical research exploring inpatient staff members’ perspectives concerning various aspects of this work [7], including collaboration and communication between staff during milieu therapeutic interventions.

The level of participation in activities, staff interactions and socialization processes involving staff members is pivotal in promoting knowledge and providing experience [6]. Milieu therapy adds collective processes and common structures to the routine activities on a day-to-day basis [8]. A therapeutic milieu is based on mutual respect, building upon positive and resilient relationships, including clinician-to-clinician relationships. Staff members use the qualities of those relations to improve the health of patients [9]. Therefore, teamwork and collaboration between inpatient staff members are important to bolster and complement therapeutic interventions for EDs [5]. The most important daily milieu therapeutic activity on inpatient EDUs occur during *mealtimes.* Mealtimes on these units are complex, involving a range of activities during which staff collaborate to ensure a supportive atmosphere to normalize patients’ relationship with food, and subsequently to gain weight. However, mealtimes are often perceived by staff as stressful and emotionally demanding. Establishing and maintaining therapeutic relationships with patients at the individual and collective level are challenging, potentially further challenged by ambivalence to treatment and a lack of motivation for recovery among patients [7, 10]. This ambivalence can surface as significant tension, which is particularly evident during mealtimes due to patients’ extreme fear of weight gain and negative emotions associated with food and eating [11, 12]. A struggle for control between staff and patients may ensue during meals, sometimes described as a battleground indicative of a “us” and “them” culture [13]. Staff members’ main responsibilities during meals include food preparation, monitoring food intake and supporting and supervising patients, which should ideally occur in a positive and relaxed atmosphere. Additionally, meals provide valuable therapeutic opportunities to challenge patients’ eating difficulties in a safe and structured environment, and to concurrently facilitate a normal mealtime conversation in a friendly atmosphere [13, 14].

Mealtime management training is percieved as essential for new staff members. Training may include role play of mealtime scenarios, with demonstration of skillful, good teamwork and collegial support important to ensure the smooth running of meals [13]. Staff collaboration whilst performing meal support is a shared task conducted in a shared workspace, i.e., the unit’s dining room. We have previously reported findings, based on video observations of meals, that staff’s meal management is informed by two main interactional scripts to facilitate a) normalized eating behavior and b) meal completion during the allotted time frame to perform the various activities within a meal [15]. Mealtime management is about balancing these two scripts, which is primarily learned through direct participation in meals, and on-the-job training. The present article builds and elaborates on this finding, eliciting involved collaborative strategies.

In healthcare, collaborative work is usually mediated through physical objects. The physical work environment becomes a medium for mediating activities of staff members. Additionally, nonverbal communication is a common way of exchanging information [1]. However, real-world teamwork and staff collaboration is under - researched and under - theorized [16]. This is specifically the case for collaboration that occurs in activities that combines therapeutic and everyday dimensions to achieve treatment goals.

Consequently, the aim of the paper is to explore staff’s perspectives on teamwork during mealtimes on inpatient EDUs. Specifically, we investigated the collaborative strategies employed to support core therapeutic goals of meal completion and normalized eating behavior, while concurrently maintaining a supportive, friendly atmosphere during mealtimes. To this aim, we applied perspectives from cultural historical activity theory (CHAT) as a useful analytical framework to explore the actual “how to” of complex practices within a specific institutional setting [17].

## Methods

This investigation is a descriptive, exploratory, and qualitative study. An exploratory design was deemed appropriate because very little is currently known about teamwork and staff collaboration during mealtimes on inpatient EDUs. The empirical findings reported in this paper is drawn from interview data, which is part of a larger data corpus of video recorded observations of mealtimes and interviews with participating staff members, focusing on teamwork and interaction during mealtimes on EDUs. Data from video-recorded observations exploring the internal structure in a meal and from interviews and video observations exploring staff behavior in staff – patient interactions have been published elsewhere [14, 15].

### Participants

Participants were recruited in an information meeting at the unit, attended by the researcher and one of the co-authors. Twenty staff members, 18 females and two males, consented to participate in the study. They were employed as milieu therapists. The male-to-female ratio is reasonably representative for the staff at the time of the interviews. Mean age of participants was 41 years (range: 26–52). The average work experience at the EDU was 4.7 years (range 0.5–5.5). There is no formal meal support training for staff on this unit. Consequently, their experience with meal support is connected to work experience, exposure and on – the – job training. At a minimum, all staff members held a bachelor’s degree, specifically, nine were registered nurses (RN) and 11 had various professional backgrounds, e.g., social workers, child welfare officers, or similar.

### Setting

This study was conducted on a 12-bed psychiatric inpatient EDU in Norway, specialized in the treatment of adult patients with a severe ED. The unit is part of a department offering specialized ED treatment for the entire south-eastern region of Norway, with a catchment area population of 2, 8 million. The majority of patients admitted to this unit are females diagnosed with AN, with a mean body mass index of 14.8 at admission [18]. As part of the treatment, all patients participate in the collective activity to “eat 4 main meals per day,” which includes breakfast, lunch, dinner and an evening meal. Each meal lasts 30 min. All patients have their own individual meal plan based upon a “basic meal plan” determined by a dietician. There are two tables in the dining room, and seating depends on the physical condition of the patient, required degree of mealtime support, and whether patients receive pre-prepared meals or serve themselves from a buffet. Staff members are present at both tables throughout the meal to observe and supervise patients.

### Data collection

Data were collected from April 2013 to June 2013, via 20 semi-structured interviews with staff members. All interviewees had participated in meals subject to observation prior to the interview. The videotaped meal was used as a starting point for the conversation. An interview guide (available on request) with questions to elicit various aspects of interaction and collaboration between participants and of their experiences during the meal assisted the interviews. All interviews were conducted as soon as possible following the meal, preferably before the next meal occurred, to help the participant to focus on and share reflections about the meal in question, and to remember the meal as accurately as possible. Most interviews lasted between 20 and 40 min. All interviews were conducted by the first author, audio taped and transcribed verbatim.

### Ethics

The study was guided by sound ethical conduct, adhering to the principles of informed consent, anonymity, and the right to withdraw from the study at any time. Approval from the Data Protection Committee at Oslo University Hospital, Oslo, Norway was obtained before collection of empirical data commenced (approval number 2013/1157). The data were managed and securely stored in a secured database, according to laws and guidelines regulating research. When presenting raw material, either in written or verbal form, principles for de-identification were followed, such as omitting information which could lead to the identification of actual persons (patients and staff members).

Participants were recruited at an informational meeting at the unit a few weeks before data gathering commenced. Potential participants were informed about the study, background and procedures for data gathering, and had the opportunity to ask questions. Written information about the study, including consent forms, was handed out during the meeting. Staff members signed and delivered the forms to an independent contact person at the unit after the meeting, to avoid any potential pressure by the researchers to participate.

Patients were carefully informed about the study before data collection, specifically emphasizing the study focus’ being on staff’. All patients signed written forms, consenting to be present during the meals, which were subject to observation. Only one patient chose to refrain from participating in the observed meals. This patient ate meals in a different room, and received the same treatment and level of support as usual. All patients were considered physically stable enough by their treatment teams to participate in mealtimes subject to video observation.

### Analysis

#### Cultural historical activity theory (CHAT) as a resource for analysis

Professional practice is often portrayed as acultural and static [16]. There is little focus on the environments within which milieu therapeutic interventions is delivered. To understand, and possibly improve or change a practice, analyzing its current and historical status is important [19]. We therefore employed a CHAT framework to better understand the nature of collaborative complexity and its implications for current approaches to collaboration during mealtimes. CHAT provides a sociocultural framework to examine how people do things together in complex environments, emphasizing collective knowledge and expertise as the dominant source of learning [20]. CHAT’s conceptual and methodological resources are therefore amenable to exploring collaborative activities in a workplace. Although previously used to elaborate workplace complexities, including healthcare practices [16, 21–23] an investigation of specific tension-laden activities such as anxiety- provoking mealtimes on an EDU, could inform the on-the-job training necessary for such interactions.

CHAT conceptualizes *activities* as motive driven [19], embedded in the “object oriented, collective, and culturally-mediated human activity” ([24] p:964]). Activities consist of both conscious strings of actions, and routinized scripts [25, 26]. The third generation model of CHAT involves at least two interacting activity systems, with a shared object, where interacting components of subject, object, tools, division of labor, community and rules focusing on a shared object points to outcomes of an activity [24]. Our activity “mealtimes on inpatient EDUs” involves staff members (*subjects*) working collaboratively to manage a meal (*shared object*) to realize the objective of the activity. The two main goals of this activity are 1) meal completion [4], and 2) facilitation of normalized eating behavior [15]. Mealtime activities are mediated by a set of *tools*, used by staff members during the meal to influence, or *mediate* interaction between subjects. Tools can be either physical/material, like books or a computer, or cultural/symbolic, like language or traditions. Formal or informal *rules,* e.g. that a meal lasts for 30 min, regulate the subject’s actions as well as relations with other participants in the activity. The *community* consists of people who share an interest in, and are involved with, the same object, i.e. the staff members participating in the meal. *Division of labor* describes division of the roles and tasks of the various staff members participating in the meal [24, 25, 27]. In this perspective, collaborative processes between staff members can be defined as ongoing strings of actions between the subject and the community, engaging a shared object [28].

All interviews were uploaded into qualitative data management software [29]. We conducted a deductive or “top-down” thematic analysis, using the CHAT framework. In line with the analytic foci: exploring how staff members (subjects) collaborated during the meal to achieve the shared object (mealtime management), the data was analyzed using three key concepts of CHAT [30] as a main analytic focus; tools, rules and division of labor. The data analysis consisted of three steps: 1) initial reading and screening of the material for passages concerning staff collaboration, 2) coding of relevant material, using the named components as main codes, 3) systematic revision of coding, and 4) development of broader themes.

The analyses were checked by an independent rater, who reviewed and coded half (10) of the transcripts independently. Subsequently, themes were compared and modified to reach consensus between the independent rater and the lead researcher. The two co-authors were also involved in discussion concerning the themes to arrive at final consensus.

### Findings

In line with the aim of this article, we particularly focused on staff perspectives concerning collaborative strategies used during mealtimes. The analysis yielded three themes.

1. *Strategic seating arrangements mediates division of labor between staff members*
2. *The use of verbal and nonverbal communication as collaborative tools*
3. *The importance of experience as a collaborative resource*

To illustrate the findings, we present extracts from the data material for each theme, along with an empirical analysis. Quotes are translated from Norwegian to English, as directly as possible, to retain their meaning. Names on staff members are fictional to protect anonymity. Comments in brackets in the quotes are the authors’ additions to increase readability.

#### Strategic seating arrangements mediates division of labor between staff members

At the start of the meal, when getting seated, staff members described how they deliberately chose opposite middle seats at the table, making sure they could face each other.

*You place yourself strategically. When my colleague had sat down by the window, it’s normal that she takes [responsibility for] the patient next to her. And it’s very normal that I take the patients seated close to me. Do you see what I mean? (Paul, child welfare officer).*

This seating pattern was described as a deliberate, strategic choice for division of labor between staff members, particularly with regard to operating rules about who was responsible for which patient during a meal. At the same time, how seating informed task allocation was viewed as something that “just happens”. As explained by Paul, dividing tasks based upon where they were seated was described as the natural division of labor by staff members*,* normally not discussed prior to, or during the meal, indicating that this is a well-established rule. Exceptions were if staff members knew beforehand that some patients were struggling, or that they had been given specific tasks by team members not participating in the meal: *“I don’t normally say that [role allocation] out loud unless we have a patient for whom things are very, very difficult” (Caroline, nurse).* If that was the case, those tasks were explicitly divided between staff members before the meal began*.*

Tensions could arise if the seating pattern for some reason was changed, if staff members were seated far apart from each other, or on the same side of the table. One of the participants described a situation where her colleague was late, and ended up next to her, at the end of the table:

*Then she doesn’t really need to be there. She is seated at the end of the table, right next to me, but far away from the patients. We can’t look at each other and the patients [simultaneously], or establish a dialogue with the patients (Emma, nurse).*

Being on the same side of the table disrupted the normal pattern of division of labor between staff members, and constrained the opportunity for exchanges during the meal. Also, the interaction between staff and patients was hindered. However, resolving this during the meal seemed challenging, especially if they wanted to avoid disturbing the patients.

#### The use of verbal and nonverbal communication as collaborative tools

In explaining how staff collaborated during a meal, the use of communicative tools was a central theme. In particular, this involved when and how to communicate using verbal or nonverbal strategies. Staff members revealed rules or conventions guiding staff collaboration, concerning what should or should not be said out loud during the meal. In the interviews, most staff members differentiated carefully between “stuff that can be said out loud” and “*stuff that can’t*”. When interacting during a meal, the form of communication chosen largely depended on what they needed to communicate about. Staff members used verbal communication for practical issues, like informing colleagues about their whereabouts during a meal, or if they needed to fetch something from the kitchen. Karen (a nurse) puts it like this: “When it’s about harmless things, we speak openly. Do you sit here while…(I briefly leave the room). Sometimes we help ourselves to our food. And then we speak openly about that.” Staff preferred speaking as openly and transparently as deemed possible about practical issues. They also used verbal communication when they collaborated to establish conversation about everyday topics. However, when they needed to communicate with each other about specific issues concerning patients, they mostly chose nonverbal strategies, particularly visual communication. Below, Susan (a nurse) describes how she uses eye contact to nonverbally communicate with her colleague.

*(…) we see things from different angles based on where we are sitting, we can see things others don’t. As if I was asking: did you get that? (…) I try to show her (with my eyes) that something has happened. Or just that I see it. If you have seen it, if you are two that have seen it, you can support each other, if there is an incident (Susan, nurse).*

As illustrated above, this non-verbal or “quiet” tool was used for various purposes. Commonly, staff members used eye contact to check for agreement or to offer support. Eye contact was also used as a coordination mechanism, to send some sort of message, for example when making a colleague aware that a patient was struggling. When establishing a connection by briefly looking at each other, staff members could then choose how to proceed, depending on the situation.

Although staff members described using a colleague’s “look” as a confirmation of mutual understanding, some expressed uncertainty concerning whether this assumption was correct, and whether they were indeed able to communicate their intended message via eye contact only.

*We had eye contact when one of the patients started talking a lot, and we both noticed that we began to withdraw from the conversation. And just got quieter. We looked at each other and nodded. In my head it confirmed that it was ok to end this now [the conversation]. But I don’t know what my colleague was thinking. But I just assumed it. That’s what happened anyhow (Kim, nurse).*

Kim describes an example of ever-present uncertainty, whether they achieved a shared interpretation of their “looks”. For that reason, other staff members described being a bit more cautious when using non-verbal communication.

#### The importance of experience as a collaborative resource

Experience, both with EDs and working with each other, was used as resource by staff members when managing the meal. At the unit, most staff members were experienced in the treatment of EDs, and had worked together for several years. They knew each other well, and had an extensive knowledge of working with this patient group. *“It is a bit like this: all of us have worked here for a long time. You know what the other is thinking without talking”.* They counted on the other person, and expected her or him to think alike. If a staff member viewed her colleagues as being experienced, this affected how they communicated and worked together during a meal.

*I know that if I sat together with someone from my work team, someone I trust blindly, that I work with all the time. That I know I communicate well with. I can’t remember the last time I said – you look after her, and I will look after her (Sarah, child welfare officer).*

Sarah exemplifies how being with a more experienced colleague felt safer, and how experiences informed staff members’ expectations to each other. They trusted the other to know the “system”, the unspoken rules and structures. Knowing the other person well made verbal communication almost unnecessary. However, if one of the staff members was less experienced, or was a student/temporal staff, the most experienced automatically assumed more responsibility.

*If it was a temporary (or less experienced) staff member, it would clearly have made a difference in communication. I would have probably felt a need to define which of the patients I would pay extra attention to. If the staff had been a member of the staff group, it wouldn’t have. If it’s new staff, you have to check that you are mentally on the same page concerning what is to be done (Martha, social worker).*

As Martha explained above, staff adjusted their own behavior according to the colleagues’ level of experience, and then the division of labor became more explicit and less “natural”. In the excerpt below, one staff member describes how her collaboration with her colleague was affected by her feeling of being the most experienced.

*It is important to observe M (patient), who doesn’t eat well, if you don’t pay attention. It wasn’t really my task … but I had it in my peripheral vision that she should eat properly. I paid attention to how she was doing with food (…). I guess I took on a bit more than I needed to. (…) It wasn’t collaboration. I kind of instructed my colleague. (….). She is absolutely qualified, but she is newer than me (Caroline, nurse).*

As illustrated above, when there was a difference in the level of experience between participating staff members, a more instructive form replaced the collaborative nature of the relationship.

## Discussion

This study explored staff collaborative strategies used to support therapeutic goals during mealtimes on EDUs. Findings suggest that underlying rules appeared to mediate staff collaboration, regulated the division of labor and the preferred modes of communication between the staff members. Three main themes exemplify this finding, including strategic seating arrangements, verbal and nonverbal communication, and level of experience.

According to Engeström [24], the continuity in strings of actions constitute an activity, and is accounted for by the existence of habitual scripts that dictate the normal order of actions. According to our participants, the rationale for strategic seating is that it facilitates division of labor and continuity in the strings of actions included in the activity without the need to verbally communicate roles. The interviews indicated that the most important rule that influences staff collaboration, and division of labor in particular, was strategic seating. This is in line with what we found in our observations of the internal meal structure [15], where staff reserved or sought the middle seats during most of the observed meals, suggesting behavior in accordance with strong, interactive scripts. The analysis of the interviews supports the observation of scripted behavior by staff, and scripts can be a possible explanation for the seemingly implicit nature of staff collaboration during mealtimes. Engaging in routine or familiar work tasks reinforces what is already known by the community. These aspects of teamwork are important for refining procedures and performing tasks with a minimum of conscious thought [31]. However, Engeström [24] argues that scripted behavior does not include motive, a key theme within CHAT. The motive gives continuity to the script as embedded in the object of activity, in our case, mealtime management [ibid]. The knowledge in use in professional practice is selected, made relevant and organized according to the motives that form practice [17]. Staff members’ scripted behavior when collaborating during meals can thus be viewed as more than a habit, and rather a product of historically accumulated experience of how best to perform various actions constituting mealtime management. Additionally, seating arrangements are part of the physical environment, and can consequently be viewed as a physical artifact that facilitates the division of labor and reduces the need for explicit articulation efforts. Multiple people can thus easily integrate their contribution through the use of physical artifacts and the shared workspace [1].

Normal, scripted flow of interaction between subjects performing an activity can be described as *coordination* [32]. Unintentional deviations from the script can cause tensions and dis-coordination in interaction between staff leading to disintegration and confusion [ibid]. In our findings, this was particularly evident when there were deviations from the strategic seating script, leading to tensions between staff. Relaxing such tensions was not possible during the meal itself, due the complexity of the situation. This underlines the importance of staff discussing interactions and collaborative strategies used during the meal outside of the dining room, and emphasize the need for specific mealtime training, as suggested by Long et al. [13]. Resolving tensions in observed deviations has a potential for learning and can be a resource for on the job training [33].

Our analysis suggests that nonverbal communication, eye contact in particular, served as a key collaborative strategy for the participants in our study. When people work in close physical proximity, non-verbal communication can become a vital and efficient way of exchanging information [1]. We found that during many staff interactions, eye contact was preferred over verbal communication as a tool to exchange information and to build shared situational awareness amongst the staff group. CHAT views tools as adopted and crafted over time to mediate goal-directed activity. Tool development is shaped by the needs, values and norms of the culture in which they are created and used [19]. Due to the inherent complexity of a mealtime situation, where several activities have to be performed simultaneously, nonverbal communication might enable staff members to continue with one activity, e.g. keep a conversation going with patients, and at the same time communicate via eye contact. However, staff members did express some concern regarding the efficacy of this strategy, and feared that misunderstandings might occur. The meaning making collectively constructed in these interactions [32] might be challenged by the nonverbal nature of the interaction. Such non-verbal communication adds challenges to required workplace learning to manage a meal in this specialized EDU setting. Also, from an ethical perspective, patients’ experiences with this form of staff communication need to be further explored.

Experience level was also identified as an important factor that influenced collaboration between the individual staff member and their colleagues during the course of a meal. The significance of staff experience when performing mealtime management is also highlighted by Long et al. [13], in particular, the support from experienced staff to newer staff members. Learning the local practice and operating scripts requires time and experience, and can be explained by the fact that this type of work in a seemingly everyday situation, i.e., eating meals, is also characterized by a high degree of expertise which is also characteristic for specialized knowledge practices. According to Engeström [34], expertise can be viewed as a collective rather than individual accomplishment, “constructed in encounters and exchanges between people and their artifacts” (p 2). Our analysis showed that subjects’ level of collective expertise in a specific meal, affected how the collaboration between subjects played out. Shared experiences create collaborative intentionality capital within a team, improve performance, and provide team members with a common platform for learning and action [32]. Foot [25] suggests that expertise may function as a secondary artifact that influences how subjects employ primary tools. Susi [35] states that tools and tool use can only be understood when considered in relation to their users and the context of their use. She proposes that experience-based knowledge can be viewed as some type of psychological artifact. This type of knowledge is an important element to understand how a practice works, and being able to operate it smoothly. Also, experience with working with co-presence may contribute when sharing information that is difficult to transfer. The sample in this study consisted of different healthcare professionals employed as milieu therapist. It could have been expected that differences between the professions were reflected in the findings, particularly in relation to experience. However, this was not the case in our study. Whether there are differences between the various disciplines involved in mealtime support should be further explored.

Mealtime training to prepare staff before meals has previously been underlined as important [13]. “Learning by doing” and on-the-job training is essential in practices where underlying theoretical and practical knowledge is relatively thin [29]. Close guidance, through supervised participation, is important for learning knowledge that would be difficult to understand without the assistance of a more knowledgeable partner [36]. The nature of the knowledge used in mealtime management interactions suggests that practice itself is crucial in learning the necessary skills [31]. Our findings suggest that it is important to convey expert knowledge and operating routines to staff when conducting such training.

### Strengths and weaknesses of the study

Although qualitative methods potentially do not form a representative view of the topic, qualitative data is valuable when we know little about the phenomenon being studied, particularly at a micro-level, which is the case in this study. The first and second authors of this paper hold extensive clinical experience with treating ED patients. The first author has several years of experience participating in meals at the EDU where data was collected. This was an advantage that eased the researcher-clinician interaction during data collection. The first author’s knowledge about the topic and of the organization may have influenced the study questions, and staff responses, in various ways. Participants may have unconsciously withheld information they assumed the interviewer was aware of. At the same time, the researcher’s knowledge may have helped to ask the “right” questions, thus acting as a driver for new insights and elaboration of data. Furthermore, within a socio cultural paradigm, the research interview is viewed as a collaborative process where knowledge is co-constructed between the researcher and the research participant [37].

The lack of patients’ perspective is another potential weakness in this article. However, the study focus was on staff interaction, and we acknowledge the need for further examination of how patients view interactions and collaborations between staff members in EDUs. CHAT provided a structure for analysis. Viewing staff members as drivers of activity and focusing on meditating factors offered a framework to observe and analyze the current state of the activity systems. Additionally, CHAT underlines the value of contradictions and variation for development of practice, rather than seeking strict conformity of “best practice” [22]. Furthermore, this allows for exploring collaboration, and we would like to point out huge potential for knowledge sharing and workplace learning in such interactions. However, it is important to keep an open mind when collecting and analyzing data using an a priori framework.

## Conclusion

In this study, we found that mealtime collaborative strategies on inpatient EDUs were mainly of non-verbal nature. Therefore, exploring how *tools*, *rules* and *division of labor* plays out help understand collaboration and activities in a shared workspace. Command of these elements of the activities is important, and makes level of and access to collective experience as important premises for how staff collaboration played out throughout a meal. These findings can contribute to enhanced insight in everyday activities, supporting treatment goals that are common nursing activities in interdisciplinary collaboration.

Greater awareness about how collegial collaboration is practiced during a key therapeutic intervention may help staff members to learn routines and regulate scripts for mealtime practices. Furthermore, increased knowledge among clinicians regarding collaborative procedures is important to assess and critically appraise what are often implicit routines, and can be used when training new staff. Also, knowledge about how meals on EDUs are managed by staff is important to develop specific interventions to better support patients during mealtimes.

## Abbreviations

*AN*: *Anorexia Nervosa*
CHAT: Cultural historical activity theory
ED: Eating disorder
EDU: Eating disorder unit
RN: Registered nurse
